# Short‐ and long‐term evolution in our arms race with cancer: Why the war on cancer is winnable

**DOI:** 10.1111/eva.12612

**Published:** 2018-03-08

**Authors:** Jay A. Rosenheim

**Affiliations:** ^1^ Department of Entomology and Nematology and Center for Population Biology, University of California Davis Davis CA USA

**Keywords:** arms race, cancer, cultural evolution, somatic selection, war on cancer

## Abstract

Human society is engaged in an arms race against cancer, which pits one evolutionary process—human cultural evolution as we develop novel cancer therapies—against another evolutionary process—the ability of oncogenic selection operating among cancer cells to select for lineages that are resistant to our therapies. Cancer cells have a powerful ability to evolve resistance over the short term, leading to patient relapse following an initial period of apparent treatment efficacy. However, we are the beneficiaries of a fundamental asymmetry in our arms race against cancer: Whereas our cultural evolution is a long‐term and continuous process, resistance evolution in cancer cells operates only over the short term and is discontinuous — all resistance adaptations are lost each time a cancer patient dies. Thus, our cultural adaptations are permanent, whereas cancer's genetic adaptations are ephemeral. Consequently, over the long term, there is good reason to expect that we will emerge as the winners in our war against cancer.

## 1 INTRODUCTION

The last two decades have seen a remarkable acceleration in the introduction of new drugs and other innovative therapies for the treatment of cancer. Most notably, these have included targeted chemotherapeutic drugs, whose enhanced selectivity of action can spare patients the devastating side effects of broad‐spectrum cytotoxic agents, and novel immunotherapies, including both immune checkpoint blockades and methods involving genetic manipulation of a patient's own T cells (Klebanoff, Rosenberg, & Restifo, 2016; Lopez & Banerji, 2017; Pardoll, 2012). The excitement surrounding these advances is, however, universally tempered by the recognition that cancer cells have demonstrated a powerful ability to evolve resistance against virtually all classes of therapies other than complete surgical excision (Faltas et al., 2016; Gottesman, Lavi, Hall, & Gillet, 2016). After the dramatic initial successes of targeted chemotherapies for treatment of chronic myeloid leukemia, failures of later‐introduced drugs have been profoundly disappointing (Gillies, Verduzco, & Gatenby, 2012; Horne et al., 2012; Schmitt, Loeb, & Salk, 2016). Just as had been observed with the less selective cytotoxic chemotherapeutic drugs (Chabner & Roberts, 2005), resistance has been shown to evolve through many mechanisms, including mutational changes in the target, downstream activation of inhibited pathways, substitution of alternate pathways for growth activation, or overexpression of efflux pumps (Gillies et al., 2012; Gottesman et al., 2016; Schmitt et al., 2016). Failures of immunotherapies due to selection for resistant cancer lineages are similarly being reported (Restifo, Smyth, & Snyder, 2016). This has called into question the future course of our “war on cancer”—the effort to find a cure for cancer that was metaphorically declared in 1971 with the signing of the U.S. National Cancer Act (Marshall, 2011)—and whether a definitive victory is a realistic expectation. We are engaged in an evolutionary arms race with cancer; can we anticipate the eventual outcome?

Here, I argue that to answer this question, it is critical to consider the timescales of the evolutionary processes involved, including the oncogenic (“intrasomatic”) selection in cancer cells on the one hand, versus the process of cultural evolution in human populations, through which scientific and technological knowledge is accumulated over time (Mesoudi et al., 2013), on the other hand. My thesis is that our ability to build an ever‐expanding set of anticancer tactics through a long‐term and continuous process of cultural evolution gives us a key advantage over cancer cells, whose resistance adaptations are retained only over the short term, being lost every time a human cancer host dies or the cancer is cured. Our cultural adaptations are permanent; cancer's genetic adaptations are ephemeral. Thus, we are the beneficiaries of a fundamental asymmetry in our arms race with cancer, making the war on cancer winnable.

## 2 ARMS RACES BETWEEN HUMAN POPULATIONS AND INJURIOUS ORGANISMS

To frame this argument, it is helpful to begin with more typical evolutionary arms races that occur between human populations and various injurious organisms whose populations we perennially attempt to suppress. Injurious organisms include those that attack us directly (human parasites and pathogens); organisms that vector pathogens to human hosts; organisms that compete with our crops (weeds) or that directly attack our crops or domesticated animals; and organisms that attack or infest our homes. To suppress these disease or pest populations, we deploy a huge array of drugs, pesticides, and other suppressive measures on a global scale (REX Consortium 2012). In each case, this sets in motion an evolutionary arms race, pitting one long‐term evolutionary process (human cultural evolution, as we invent new control tactics) against another long‐term evolutionary process (evolution of resistance by natural selection in the populations of the injurious organisms). There is a symmetry to these arms races: On both sides, there are continuous processes in which key innovations (cultural adaptations or genetic adaptations) can be retained permanently, resulting in what Daniel Dennett (1995) has called the “accumulation of design.” The power of evolution by natural selection operating across many generations is, as every evolutionary biologist knows, immense; thus, it should come as no surprise that we face a stiff challenge in our attempts to outpace its effects. Ever‐worsening problems with drug‐resistant bacteria and viruses, and pesticide‐resistant weeds, crop pests, and insect vectors of disease, show that the outcomes of these balanced evolutionary processes are uncertain (Arts & Hazuda, 2012; Goldberg, Siliciano, & Jacobs, 2012; Kennedy & Read, 2017; REX Consortium, 2012). It is hard to know if we will be able to stay one step ahead, and predictions in the realm of antibiotic resistance are becoming increasingly alarming (CDC 2013; Davies & Davies, 2010; Dheda et al., 2014).

In attempting to control populations of injurious organisms, we also may confront the tragedy of the commons dynamic. Susceptibility in a population of an injurious organism to an effective drug or pesticide is a genetic resource that is shared by society and that can be depleted. Access to that resource cannot, however, be fully controlled by any individual. Thus, society encourages patients and physicians to use antibiotics conservatively and implores farmers to use pesticides sparingly, but in both cases the prospect of short‐term personal gains by heavy users may lead to a more rapid loss of susceptibility than society as a whole would view as optimal (Hardin, 1968; Porco et al., 2012).

## 3 ARMS RACES BETWEEN HUMAN POPULATIONS AND CANCEROUS CELL LINES

We also find ourselves battling another unwanted replicator: Our own somatic cell lines that have thrown off the normal restraints on their proliferation. Just as is the case with our efforts to control disease and pest organisms, our work to develop cancer treatments unfolds as a continuous, long‐term process of cultural evolution. The outcome of this process is an ever‐expanding arsenal of anticancer drugs (Barlas, 2016).

In the short term, cancerous cell lines have an outsized ability to evolve resistance to chemotherapies (Greaves & Maley, 2012; Nowell, 1976). This is fueled by what is often a very high level of genetic diversity produced by mutation in cancer cells. The mutation rate observed in healthy somatic cell lineages is substantially higher than that observed in germ cell lineages (Milholland et al., 2017), and histologically and physiologically normal somatic cells can, as a result, accumulate very large mutational burdens (Martincorena et al., 2015). On top of this, some cancer cells exhibit an insidious form of positive feedback that leads to an acceleration of their genetic and epigenetic instability: As cancer cells acquire mutations, genes involved in DNA replication, DNA repair, and the mitotic machinery may become damaged, driving the underlying mutation rate still higher (Alexandrov et al., 2013; Hanahan & Weinberg, 2011; Loeb, 2011). In addition, deregulated DNA replication and uncontrolled cell division associated with oncogenic transformation can cause a host of problems, including telomere erosion and associated breakage–fusion–bridge cycles, nucleoside imbalances, and DNA replication stress, all of which generate additional mutations, including many chromosomal mutations (Burrell, McGranahan, Bartek, & Swanton, 2013; Chiba et al., 2017; Maciejowski & de Lange, 2017; Mathews, 2015; Ren et al., 2017). An escalating mutation rate speeds the accrual of still more mutations in genes whose function is to safeguard the integrity of the genetic material. The result can be an explosive evolutionary potential over the short term, such that cancer cells become so heterogeneous and malleable that mutants conferring resistance to virtually any therapy often exist before the therapy is initiated (Faltas et al., 2016; Greaves & Maley, 2012; Loeb, 2011; Schmitt et al., 2016). Rapid evolution of resistance is thus a major obstacle to establishing effective and durable cancer therapies and is the primary cause of disease relapses for cancer patients (Aktipis, Kwan, Johnson, Neuberg, & Maley, 2011; Greaves & Maley, 2012; Nowell, 1976; Thomas et al., 2013).

This frighteningly potent short‐term evolutionary potential of cancer cells is not, however, matched by a similarly potent long‐term evolutionary potential. In fact, cancer cells have no long‐term evolutionary potential at all. Rather, a defining feature of oncogenic or intrasomatic selection is that it is constrained to act as a short‐term, discontinuous, or episodic process, with each episode confined to events occurring within a single host, and ending abruptly when that host dies (from whatever cause) or the cancer is cured, eradicating all cancer cells (Aktipis & Nesse, 2013; Arnal et al., 2015; Crespi & Summers, 2005; Ewald & Swain Ewald, 2012; Haig, 2015 Merlo, Pepper, Reid, & Maley, 2006; Ujvari, Papenfuss, & Belov, 2016b). Resistance adaptations, like any other adaptations that might evolve in cancers, thus cannot persist beyond the death of each host—there is simply no way for cancer cell lines (or, at least, noninfectious cancer cell lines—see below) to pass adaptations to a new host. Thus, cancer evolution must start from scratch with each new host. Every cancer begins its evolutionary arms race against our control measures as a naïve combatant.

Note that this line of reasoning also holds for cancer driver gene mutations that are hereditary. Although a copy of a hereditary cancer driver gene that is present in a tumor cell might mutate, conferring resistance to a chemotherapeutic drug, this mutated version of the gene has no opportunity to be passed to offspring of the cancer patient, because it is found only in the patient's tumor cells, which are somatic cell lineages, and not in the patient's germ cells.

Despite the decidedly mixed record of success for newly introduced cancer drugs, we are now beginning to see how this asymmetry in our arms race against cancer—a continuous process of cultural evolution pitted against a discontinuous process of oncogenic selection—can give us a decisive advantage over the long haul. Governmental and, increasingly, private‐sector investment in discovering new cancer treatments is yielding a rapidly expanding set of chemotherapeutic agents, immunotherapies, and other therapies that are contributing to our ability to suppress cancers (Barlas, 2016). Newly introduced drugs can be useful in at least three ways. First, even if a new drug is no more effective than previously developed drugs, it can be used in a sequence following other drugs to extend the life of the patient, providing one more cycle of temporary efficacy (Soverini et al., 2014). A series of temporary disease remissions can add up to a substantial extension of life for many patients. Second, a new drug may offer improvements in either efficacy or in selectivity, offering a lessening of side effects. Third, and perhaps most significantly, by expanding the set of effective options available to clinical oncologists, new drugs create possibilities for combinatorial therapies.

Combinatorial therapies have emerged as the main generators of cancer cures. They offer two distinct kinds of advantages: First, drug combinations may kill a greater proportion of tumor cells than either drug used singly (Lopez & Banerji, 2017). For instance, enhanced efficacy may be observed when an antitumor vaccine is combined with an immune checkpoint blockade drug: Antitumor vaccines induce immunogenic cell death, generating a de novo immune response that is then coupled with an immune checkpoint blockade drug, which ensures that the immune response is not subsequently downregulated by tumor cells (Vilgelm, Johnson, & Richmond, 2016). Second, combinatorial therapies are increasingly recognized as our most powerful means of preventing cancer cells from evolving resistance (Hanahan, 2014; Kaiser, 2011; Loeb, 2011; Lopez & Banerji, 2017). In fact, combinatorial strategies appear to retard resistance evolution in diverse settings where resistance evolution is an especially acute concern (Goldberg et al., 2012; Zur Wiesch, Kouyos, Engelstädter, Regoes, & Bonhoeffer, 2011): Drug cocktails have provided a means of coping with drug‐resistant bacterial and viral pathogens (e.g., *Mycobacterium tuberculosis*, Gandhi et al., 2010; *H. pylori*, Hu, Zhu, & Lu, 2017; HIV, Arts & Hazuda, 2012), and toxin combinations can effectively slow resistance evolution in insect herbivores feeding on transgenic crop plants (Carrière, Fabrick, & Tabashnik, 2016). Resistance evolution against vaccines appears to be rare largely because vaccines induce immune responses against multiple therapeutic targets on the pathogen simultaneously, thus generating the multiple redundant pathways to pathogen killing that are at the heart of the combination strategy (Kennedy & Read, 2017).

The logic underlying the efficacy of combinations is simple (REX Consortium 2012): Returning to the case of cancer cells, if clones resistant to any single drug are present at low frequency (say, 1 × 10^−5^), then given the huge number of cells present in even small tumors (frequently >10^8^; Loeb, 2011), we expect >10^3^ cells to be resistant, and use of a single drug is bound to fail, as has sadly been abundantly confirmed in practice. But combining two drugs raises the bar: If each drug can kill susceptible tumor cells using an independent mode of action, then only clones harboring two mutations can survive, and such clones are expected to be rare: (1 × 10^−5^) × (1 × 10^−5^) = (1 × 10^−10^). This is true regardless of how diverse the mutations conferring resistance to a particular drug might be (and they are often very diverse: e.g., Khorashad et al., 2013). By extending this to three or four drugs, we make it increasingly unlikely that a full set of requisite resistance‐conferring mutations will be found in any single cancer cell. This is why many of the dramatic successes achieved in the early decades of the war on cancer—those yielding high cure rates—involved deploying multiple cytotoxic chemotherapies together (e.g., methotrexate + vincristine + 6‐mercaptopurine + prednisone, “POMP,” for childhood acute lymphoblastic leukemia; nitrogen mustard + vincristine + procarbazine + prednisone, “MOPP”, for Hodgkin's lymphoma [Chabner & Roberts, 2005], platinum + vinblastine + bleomycin, “PVB,” for testicular cancer [Einhorn, 1981]). Combinatorial therapies can still fail when mutations emerge that confer broad‐spectrum resistance (e.g., multi‐drug resistance generated by overexpression of drug efflux pumps; Yang & Fu, 2015; Gottesman et al., 2016; see also Baym, Stone, & Kishony, 2016), and combinatorial therapies also must be balanced against the severity of side effects (Lopez & Banerji, 2017) and potentially prohibitive costs. But combinatorial therapies built with increasingly selective, targeted chemotherapies and with immunotherapies (e.g., Hodi et al., 2016; Vilgelm et al., 2016; Wolchok et al., 2013) hold tremendous promise for expanding the set of curable cancers. Expanding opportunities for combinatorial therapies are some of the sweetest first fruits of the long‐term cultural evolution process playing out within the oncological research community.

Our steady progress in the development of more effective cancer therapies is also unfolding without the ethical dilemma that we would encounter if cancer therapy were to conform to the tragedy of the commons. Because resistance adaptations in cancer patients are confined to each treated individual, no patient or oncologist has ever had to ask whether a too‐aggressive treatment might produce a resistant cancer clone that could jeopardize a future patient. Difficult societal choices may still need to be made because of cost considerations, but these choices can be made knowing that the efficacy of a drug regimen in a future patient will not be influenced by how a current patient is treated.

## 4 INFECTIOUS AND PATHOGEN‐ASSOCIATED CANCERS

The line of reasoning I have been developing applies only to cancers that cannot move between hosts (noninfectious cancers) and cancers that are not associated with pathogens. But approximately 15% of all human cancers worldwide are known to be caused by pathogens (Lunn, Jahnke, & Rabkin, 2017; Plummer et al., 2016), and some observers suggest the true figure may prove to be higher (Ewald & Swain Ewald, 2012). Pathogens are more different biochemically from healthy human cells than are human tumor cells, offering greater opportunities for selective toxicity (Ewald & Swain Ewald, 2014, 2015), as well as more abundant preventive approaches to cancer (e.g., vaccination). Capitalizing on these opportunities, advances in the treatment some pathogen‐associated cancers are beginning to reduce cancer incidence and mortality (Casper & Fitzmaurice, 2016; Ewald & Swain Ewald, 2014; Global Burden of Disease Cancer Collaboration 2017; Plummer et al., 2016). But, might the evolutionary continuity within pathogen populations cause our arms race with these cancers to unfold in a qualitatively different, and more adverse, way? Although this discussion is admittedly somewhat speculative, it is useful to distinguish between different categories of possible cancer infection potential (Table 1).

**Table 1:** Categories of the infection potential of cancer^a^, and possible consequences for cancer resistance evolution to chemotherapies

Category of cancer	Implications for cancer's evolution of resistance to therapies	Taxa involved
1. Cancers unrelated to infection	Evolution of resistance only over the short term, within individual patients. No long‐term evolutionary process exists.	Human somatic cell lines
2. Cancers associated indirectly with infection, because: (a) infections cause chronic inflammation, which is oncogenic, or (b) infections suppress immune surveillance, which increases survival of cells transforming to cancer	Evolution of resistance only over the short term, but pathogen could evolve resistance to some cancer therapies over the long term. If cancer reduces pathogen fitness by truncating host longevity, then natural selection might favor reduced oncogenicity in pathogens, all other things being equal.	*Helicobacter pylori* Human immunodeficiency virus (HIV) *Plasmodium falciparum* *Toxoplasma gondii* *Trichomonas vaginalis* *Schistosoma* spp. *Opisthorchis viverrini* *Clonorchis sinensis*
3. Cancers directly caused by infection with intracellular pathogen; clonal expansion of infected cells may enhance virus fitness (i) by enhancing the replication of the pathogens or (ii) by evading host immune responses by opposing cell‐cycle arrest and apoptosis	Cancer evolves only over the short term, but the pathogen evolves over the long term. Because the cancer is an extended phenotype of the pathogen, evolution within the pathogen genome might oppose our therapeutic interventions. If pathogen fitness is reduced by malignant, metastatic cancer because they reduce host longevity, pathogen evolution might not oppose some therapies that impose stability on cancers rather than outright cures.	Epstein–Barr virus (EBV) Hepatitis B virus (HBV) Hepatitis C virus (HCV) Human papillomavirus (HPV) Human T‐lymphotropic virus type 1 (HTLV‐1) Kaposi's sarcoma‐associated herpes virus (KSHV) Merkel cell polyomavirus (MCV)
4. Infectious cancerous cell lines	Cancer evolves resistance over the short and long terms.	Unknown in human populations

a From Hu, Yang, Wu, Wong, and Fung (2012); Ewald and Swain Ewald (2012, 2013, 2014, 2015); Alibek et al. (2013); Wang et al. (2013); Graham (2015); Plummer et al. (2016); Chang et al. (2017); Lunn et al. (2017); Xie et al. (2017).

First, as already discussed, are cancers that are not associated with pathogens. These cancers have a very strong capacity for short‐term evolution, but no ability to evolve resistance in the long term, across hosts.

Second are pathogens, most of which are extracellular, that cause cancer largely by eliciting chronic inflammation or immune suppression in their host. Inflammation increases cancer risk through several pathways, including by enhancing production of reactive oxygen and nitrogen species that damage DNA and producing various proliferative signals (Alibek, Kakpenova, & Baiken, 2013; Brennan & Garrett, 2016; Ewald & Swain Ewald, 2014; Graham, 2015). Immune suppression disrupts immune surveillance of cancer, resulting in broad increases in cancer risk (e.g., *Schistosoma* spp., HIV; Ewald & Swain Ewald, 2012, 2013, 2014, 2015). The fitness of these pathogens is not directly enhanced by neoplasia in their host. Instead, they may benefit from the inflammation of host tissues, which may increase the availability of nutrients (e.g., *Helicobacter pylori*; Graham, 2015; D. Y. Graham, *personal communication*) or from the suppression of the host's immune system, which enables these parasites to establish chronic infections, thereby enhancing opportunities for pathogen transmission (e.g., *H. pylori*, Wang, Zhu, & Shao, 2013; Xie et al., 2017). The extent to which these pathogens evolve to oppose our anticancer therapies depends on the details of our intervention. If the anticancer interventions involve treatments directed against the pathogens themselves, then we face the full power of a continuous evolutionary process favoring resistance evolution. For example, antibiotic treatments for *H. pylori* are a standard treatment for gastric cancers, and not surprisingly resistant *H. pylori* are a growing clinical concern (Hu et al., 2017). If, however, we treat the cancer itself, for instance with cytotoxic agents or targeted chemotherapies, then our interests often do not conflict with the evolutionary interests of the pathogen, and thus, we should not expect pathogen evolution to oppose our cancer treatments. Pathogens that rely on creating partial immunosuppression of their hosts might, however, evolve to oppose cancer immunotherapies if enhanced immune activity is also expressed against the pathogen. In all cases, once a cancer progresses to a more malignant, metastatic state and threatens to kill the host, the evolutionary interests of the human host and the pathogen may become aligned in favoring ongoing host survival (Ewald & Swain Ewald, 2015), and the pathogens are not expected to oppose anticancer therapies.

Third are intracellular, viral pathogens for which the initial proliferation of infected host cells may enhance fitness in two ways. First, proliferation of infected cells may directly augment the replication of viral genome (Ewald & Swain Ewald, 2012, 2013). Second, in cases where host immune responses to virus‐infected cells involves the initiation of cell‐cycle arrest and programmed cell death, viruses may enhance their survival by opposing cell‐cycle arrest, with uncontrolled cell proliferation as a consequence (Chang, Moore, & Weiss, 2017). Evolution in these viral populations will, in both the short and long terms, oppose both direct antiviral treatments (e.g., in response to screen‐and‐treat programs against hepatitis B and C virus infections; Plummer et al., 2016) and anticancer treatments. We have, however, made great strides in preventing at least some of these cancers with vaccines (human papillomavirus, hepatitis B virus; Plummer et al., 2016; Lunn et al., 2017), which have proven to be much more durable in the face of pathogen evolution than have treatments targeting established infections (Kennedy & Read, 2017). Established infections of oncogenic viruses should evolve to oppose our cancer therapies, but it is unclear how readily viruses could manipulate the cancer phenotype of their host's cells to evade our treatments; empirical work on this question is needed. Furthermore, the finding that many tumor cells host only latent or “pseudolatent” viruses that are not actively replicating (Chang et al., 2017) suggests that viral evolution may not strongly oppose anticancer treatments. Once the cancer progresses to threaten host survival, the evolutionary interests of the cancer cells and the oncogenic virus are no longer well aligned (Chang et al., 2017; Ewald & Swain Ewald, 2015) and the reproductive value of viruses remaining in the host will be low; thus, oncogenic viruses may not strongly oppose treatments of metastatic cancer, especially if they arrest cancer development rather than creating an outright cure.

Finally, we have the case of contagious cancers, in which cancer cells themselves are capable of moving between different host individuals. Infectious cancers have been found in dogs, Tasmanian devils, and a diverse group of marine gastropods (Metzger & Goff, 2016; Metzger et al., 2016; Ujvari, Gatenby, & Thomas, 2016; Ujvari, Papenfuss et al., 2016), but, happily, not in human populations. An infectious human cancer would be a nightmare scenario for resistance evolution, as the powerful short‐term evolutionary potential of cancer cells would be extended to a longer‐term, continuous process. Although the genomic instability of most human cancers might not be sustainable over longer periods of time, due to catastrophic mutation accumulation (Andor, Maley, & Ji, 2017; Arnal et al., 2015), the strong mortality imposed on host populations by some newly emerged contagious cancers (Epstein et al., 2016; Metzger & Goff, 2016) shows how fortunate we are that we lack any such cell lines. Our highly polymorphic major histocompatibility complex (MHC) loci are likely our primary defense against the future emergence of such cancers (Ujvari, Gatenby et al., 2016; Ujvari, Papenfuss et al., 2016).

Thus, although the timescale of the arms race between human populations and pathogen‐associated cancers may be more symmetrical than the arms race between humans and nonpathogen‐associated cancers, the picture that emerges is not entirely bleak. If we can devise cancer therapies that reduce the extent to which our interests are orthogonal to the interests of the pathogens, we will avoid the full brunt of long‐term, evolved opposition to our interventions in pathogen populations.

## 5 CONCLUSION

The war on cancer has struggled during its first four decades to make major inroads on cancer mortality (Barlas, 2016; Global Burden of Disease Cancer Collaboration 2017; Marshall, 2011; Mukherjee, 2010). This is, to a large degree, due to cancer's prodigious short‐term ability to evolve resistance to our therapeutic interventions. But, with our ability to mount a sustained, continuous process of cultural evolution, in which every increase in our knowledge and every therapeutic tool devised is permanently retained, it was reasonable to expect that the tide would eventually turn. The Achilles’ heel of cancer is that it cannot retain its resistance‐conferring adaptations across different hosts. Whether with small, incremental steps, or large, dramatic leaps forward, the cumulative progress in our ability to treat cancer will, in the end, reveal the war on cancer to be winnable.

## CONFLICT OF INTEREST

None Declared.
